# Effects of a Two-Year Intensive Multidisciplinary Rehabilitation Program for Patients with Huntington’s Disease: a Prospective Intervention Study

**DOI:** 10.1371/currents.hd.2c56ceef7f9f8e239a59ecf2d94cddac

**Published:** 2014-11-25

**Authors:** Anu Piira, Marleen R. van Walsem, Geir Mikalsen, Lars Øie, Jan C. Frich, Synnove Knutsen

**Affiliations:** Department of Research and Development, North Norway Rehabilitation Center, Tromsø, NorwayNorth Norway Rehabilitation Center; Center for Habilitation and Rehabilitation Models and Services, Medical faculty, Dept. of Neurohabilitation, Oslo University Hospital, University of Oslo, Oslo, Norway; Department of Research and Development, North Norway Rehabilitation Center, Tromsø, Norway; North Norway Rehabilitation Center, Department of Research and Development, Tromsø, Norway; Department of Neurology, Oslo University Hospital, and Institute of Health and Society, University of Oslo, Norway; Department of Research and Development, North Norway Rehabilitation Center, Tromsø, Norway

## Abstract

Objective: To assess effects of a two year intensive, multidisciplinary rehabilitation program for patients with early- to mid-stage Huntington’s disease. Design: A prospective intervention study. Setting: One inpatient rehabilitation center in Norway. Subjects: 10 patients, with early- to mid-stage Huntington’s disease. Interventions: A two year rehabilitation program, consisting of six admissions of three weeks each, and two evaluation stays approximately three months after the third and sixth rehabilitation admission. The program focused on physical exercise, social activities, and group/teaching sessions. Main outcome measures: Standard measures for motor function, including gait and balance, cognitive function, including MMSE and UHDRS cognitive assessment, anxiety and depression, activities of daily living (ADL), health related quality of life (QoL) and Body Mass Index (BMI). Results: Six out of ten patients completed the full program. Slight, but non-significant, decline was observed for gait and balance from baseline to the evaluation stay after two years. Non-significant improvements were observed in physical QoL, anxiety and depression, and BMI. ADL-function remained stable with no significant decline. None of the cognitive measures showed a significant decline. An analysis of individual cases revealed that four out of the six participants who completed the program sustained or improved their motor function, while motor function declined in two participants. All the six patients who completed the program reported improved or stable QoL throughout the study period. Conclusion: Our findings suggest that participation in an intensive rehabilitation program is well tolerated among motivated patients with early to mid-stage HD. The findings should be interpreted with caution due to the small sample size in this study.

## Introduction

There has been a growing interest in studying effects of rehabilitation in patients with Huntington’s disease (HD). Several studies of treatment with physiotherapy suggest beneficial effects when using sensitive standard physiotherapy outcome measures^1^^,^ 2 ^,^ 3 ^,^ 4. Observational studies suggest beneficial effects of an intensive rehabilitation approach on symptom development of early to middle stage HD, though there is still a lack of randomized controlled studies in this field^5^^,^ 6 ^,^ 7 ^,^ 8 ^,^ 9.

Zinzi et al [5] described that early to mid-stage HD patients were able to preserve or improve cognitive and motor function after participating in a two year intensive multidisciplinary rehabilitation program that included six in-patient stays, of three weeks each, in a rehabilitation center^5^. Participants and their caregivers reported improvement in physical function, swallowing, balance, increased independence, mood, less apathy and improvement in social relations^6^. Piira et al. replicated Zinzi’s study, and found improved balance, gait function, physical quality of life and reduced depressive and anxiety symptoms in patients with early to middle stage HD who participated in a one year multidisciplinary rehabilitation program^7^. Another study of the effects of a three week long intensive multifunctional neurorehabiliation program in symptomatic HD patients found improvement in ADL- and motor functions^8^. The authors concluded that rehabilitation of patients with HD should be multifunctional and continuous in order to improve or maintain motor function and functional independence^8^. Finally, a pilot study of a nine months multidisciplinary rehabilitation program, consisting of weekly training sessions, home-based exercises and occupational therapy, compared with a control group of early to middle-stage HD patients, suggest that multidisciplinary rehabilitation has therapeutic benefits and is well tolerated^9^.

In 2009, The Norwegian Directorate of Health funded a pilot project to investigate effects of an intensive multidisciplinary rehabilitation program for patients with HD. The primary aim of the project was to replicate the results reported by Zinzi et al.^5^ , and results of the one year intensive multidisciplinary rehabilitation have been published^7^. We did a small-scale follow-up study to assess the effects of participation in a multidisciplinary rehabilitation program over two years. The aim of this article it to report results from the cohort of patients, with early to mid-stage HD, that participated in the two year intensive multidisciplinary rehabilitation program.

## Methods

*Subjects.* Ten of 20 HD patients who completed a one year program at the rehabilitation center in Tromsø agreed to continue for an additional year. They were already included in the program based on inclusion criteria in the beginning of the first year of the study: 1) age >18 years, 2) known genetic diagnosis of Huntington´s disease, 3) early to mid-stage HD, equivalent to stages I-III on Shoulson & Fahn rating scale, 4) no diagnoses of severe psychiatric disease, 5) no apparent severe impairment in general cognitive function at the time of first admission.

*Procedures*. Patients were recruited from the same rehabilitation center during the first year of the rehabilitation program. The project was submitted to the Regional committee for medical and health research ethics who considered this to be a clinical quality improvement project (ref. 2010/2629-7), exempt from approval by the ethics committee, and therefore referred the project to the Norwegian Social Science Data Services which granted approval (ref. 26587).

Patients were admitted in two groups of four to six persons each during 2011-2013. All patients and their family members received written and oral information, and gave their informed consent to participate in the project during the first year and consented to continue for an additional year. The first consent was considered sufficient for the second year since it was not registered as a second study. For all patients, the following demographic information was collected from the medical records at the time of the first admission: age, gender, marital status, estimated disease duration. Furthermore, baseline clinical characteristics were recorded using the standardized assessments of the* Unified Huntington´s Disease Rating Scale* (UHDRS)^10^.

*Description of the rehabilitation program*. The structure of the rehabilitation program was similar to the first year program. In the two year program each participant completed six admissions of in-patient stays of three weeks each. The program included up to eight hours of various activities during weekdays Monday to Friday, and four hours of supervised activities during the weekend. Daily activities included training with physio-, occupational- and speech therapists, group training in the gym and/or in the swimming pool. There were patient education sessions and group discussions as well. Family members were also included in the program. For a detailed description of the program see our earlier report 7. Patients and family members were followed up between admissions in order to ensure adequate local follow-up that could continue after finishing the entire rehabilitation program.

*Outcome measures*. The same outcome measures as described in our previous report were used, including the a) Timed-up-and-go test (TUG): the time the participant uses to stand up from a chair, walk 3 meters, turn around, walk back and sit down on the chair;^11^^,^ 12 b) 10-Meter Walk Test (10MWT): the participant walks 10 meters as fast as possible, while recording the time to complete; 11 ^,^ 12 c) Six Minute Walk Test (6MWT): measuring the distance (meters) the participant walks within 6 minutes; 12 ^,^ 13 d) Berg Balance Scale (BBS), consisting of 14 subtests covering various activities associated with balance control, where higher scores (max 56) indicate better balance 12 ^,^ 14 e) Activities of Balance Confidence scale (ABC), a 16-item questionnaire describing several tasks for which the participant indicates how confident they are in performing each of these tasks without losing their balance or becoming unstable with a higher score (max 100) indicating higher confidence 12 ^,^ 15 , in order to measure motor function, gait and balance^7^.

ADL function was assessed using The Barthel index, a 10-item rating scale, evaluating the level of assistance needed by a participant to perform basic activities of daily living^16^. Higher scores (max 20) indicates better performance.

General Cognitive function was measured by the *Mini Mental State Examination* (MMSE)^17^ Change in psychomotor speed and executive function was evaluated using the *UHDRS Cognitive Assessment*, comprising the following tests: a) Verbal Fluency Test containing letters F, A and S (generating as many words as possible starting with these letters), b) Stroop colour-word test, including three conditions: Stroop colour (naming colour blocks), Stroop Word (reading colour words printed in black ink), Stroop interference (naming the ink colour of incompatible colour words), c) Symbol Digit Modalities Test (SDMT) (the patient has to pair digits to assigned symbols with help of a reference key)^10^. Higher scores indicate better cognitive function on these measurements. Participants completed a 14-item self-report, *The Hospital Anxiety and Depression Scale*(HADS), in order to assess symptoms of anxiety and depression^18^^,^ 19.

Participants´ quality of life was assessed using the *Short Form-36* (SF-36) a self-reported questionnaire with two component scores, for physical and mental quality of life^20^. Furthermore, the participants’ body mass index (BMI) was recorded at the beginning and end of each admission. Participants with a BMI lower than 21 were monitored by a dietitian during the in-patient period.

*Assessments*. Gait and balance assessments were completed at the beginning and end of each admission, resulting in a total of 14 assessment points during the two year period. ABC scale, Barthel index, MMSE, HADS and SF-36 were completed at the beginning of each admission, generating a total of eight assessment points during the two year period. UHDRS Cognitive Assessment was used three times; baseline, 15 months and the evaluation after two years. All assessments were conducted by experienced and trained staff and, if possible by the same staff member during the full two year program.

*Statistical analysis.* Due to small sample size and non-normal distributions, the non-parametric Friedman’s ANOVA was used to calculate the overall mean change effect from baseline (admission 1), the 15th month evaluation and the final evaluation stay for all the variables. Confidence Intervals (CI) were calculated and reported for all measures at the three measure points. For post hoc procedures, pairwise comparisons between baseline, 15 months evaluation and the final evaluation after two years were performed using the Wilcoxon Signed Rank test. The family wise error rate was controlled for by Bonferoni correction. The SPSS software, version 21 was used for all statistical analyses. Level of significance was set at p<0.05. We will report assessments at the time of the first admission at baseline, and again at 15 months and two years at group level. Additionally, we will report on the individual baseline and final evaluation stay scores for gait, balance and quality of life for the 10 participant who entered the second year of the program for a total eight measure points.

## Results

*Characterization of the sample.* Baseline demographic and other characteristics of these 10 patients were the following: the mean age was 50.0 (SD±14.0) years with 50% (n=5) of the patients being women; 60% (n=6) was married and 90% (n=9) had children. Only one patient (10%, n=1) smoked. All patients had initiated or established an individual plan for coordinated health care prior to entering to the second year of the rehabilitation program, and 60% (n=6) had some professional home assistance. The mean BMI was 23.6 (SD±2.8). Mean time from the baseline to the two year evaluation was 783.0 days (SD±28.0). Mean symptom duration was 6.6 (SD±4.3) years and mean total functional capacity (TFC) score was 8.7 (SD±2.5).The mean scores for the UHDRS motor and behavioral scales were 47.4 (SD±9.8) and 7.4 (SD±6.9), respectively. Mean MMSE score was 23.5 (SD±4.1).

*Motor function*. Results showed a slight decline or stable function in gait (assessed by TUG, 10MWT and 6MWT) from baseline to the 15 month evaluation and to the two year evaluation (see Table 1). The mean changes in gait assessments from baseline to the evaluation after two years were small and not statistically significant: TUG + 2.5 seconds, 10MWT – 0.17 m/s and 6MWT + 4.3 meters. Minor declines in balance was detected (also shown in Table 1) with a mean change of – 1.4 points for BBS and of 8.7 points for the ABC scale. These changes were not statistically significant. ADL-function as measured by Barthel Index showed a non-significant minor change of -0.2 points from baseline to the two year evaluation.

Analysis of the individual cases showed that, among the six participants who completed the two year program, four had stable or improved gait measured by TUG from baseline to the last measurement point (case 2 -1.8 sec, case 3 -2.1 sec , case 8 -2.4 sec and case 9 +0.8 sec) (see figure 1). Two participants, cases 5 and 7, showed a clinically meaningful decline exceeding 2.98 sec^4^. Case 5 was stable until the seventh measurement point and then a sudden decline in gait at the eighth measurement point occurred (change +12.8 sec), and case 7 declined slowly over time (+7.6 sec) (See figure 1). Figure 2 illustrates that cases 7, 8, and 9 had stable gait speed measured by 10MWT (changes of -0.1, +0.1 and -0.1m/s, respectively). Cases 2, 3 and 5 experienced a slight decline in gait speed of-0.21, -0.43 and -0.23 m/s, respectively. A clinically meaningful change in 10MWT must exceed 0.34 m/s^4^. Overall two cases had stable or improved function measured by 6MWT with changes from baseline to the last measurement point of +142 m for case 3 and +82 m for case 8 (Fig.3.). A slight decline in walking distance was found for cases 2, 7 and 9 during the study period (-38, -41and -69 m, respectively). Case 5 had stable function over time but walked 50 m shorter at the last measurement point compared with baseline (shown in fig. 3). Only case 3 had a clinically meaningful change^4^.

Similar findings were found for balance. In general, BBS scores were stable or improved for four individuals throughout the study period. Changes in BBS from the baseline to the measurement were ±0; +3; ±0 and +2 points, respectively for cases 2, 5, 8 and 9. Cases 3 and 7 experienced a decline in balance (fig. 4) with changes in score from baseline to the last measurements of -3 and -6 units, respectively. Only case 7 had a clinically meaningful change in BSS that exceed 5 points^4^ .On the ABC scale, only cases 3 and 8 reported increased confidence in their own balance over time with a change in score from baseline to the last measurement of 34.9 and 2.5 units, respectively (Fig. 5). Overall, the participants who did not complete the full two year program (case 1, 4, 6 and 10) had stable balance and gait function until they left the program (shown in figures 1-5).

*Cognitive function*. The MMSE mean score showed a small improvement of +1.3 points from baseline to the evaluation after two years. The mean scores on the UHDRS Cognitive assessment also showed declines between baseline and the two year evaluation. These declines were -1.0 points for the FAS test (cognitive regulation), -3.3 points for the Stroop color naming task (psychomotor speed, automatization), -5.6 points for the Stroop interference test (psychomotor speed, cognitive inhibition), -5.0 points for the SDMT (psychomotor speed, cognitive effectiveness). The Stroop word reading task (psychomotor speed, automatization) showed virtually no change in score with a 0.3 change. None of these changes were statistically significant. The results are shown in Table 1.

*Anxiety and depression*. Although patients initially were only slightly affected by anxiety and depression, symptoms of anxiety and depression were reduced from baseline to the evaluation stay (Table 1), and showed a continuous reduction throughout the whole project.

*Quality of life*. Patients reported improvement in both the physical component score (13.0 points) and mental component score (10.3 points) from baseline to the two years evaluation (Table 1), but these changes were non-significant. Only participant 7 had a slightly lower physical QoL score from the baseline to the final assessment point change, – 3 units, and cases 2, 3, 5, 8 and 9 reported improved or stable QoL (Figure 6). Also change in Mental QoL scores for the individual cases 2, 3, 5, 7 and 8 were positive and only case 9 had a minor reduction of -2 units in QoL scores (Figure 7). The participants that did not complete the full two-year program (cases 1, 4, 6 and 10) did have improvement in QoL until they left the program (shown in figures 6 and 7).

*BMI*. Patients gained some weight during the project period, as seen by an increase in BMI of 2.4 units from baseline to evaluation. The mean BMI at the end of the evaluation stay lies slightly above the normal range (BMI 18-25) indicating a mean weight somewhat above normal.

**Figure:**
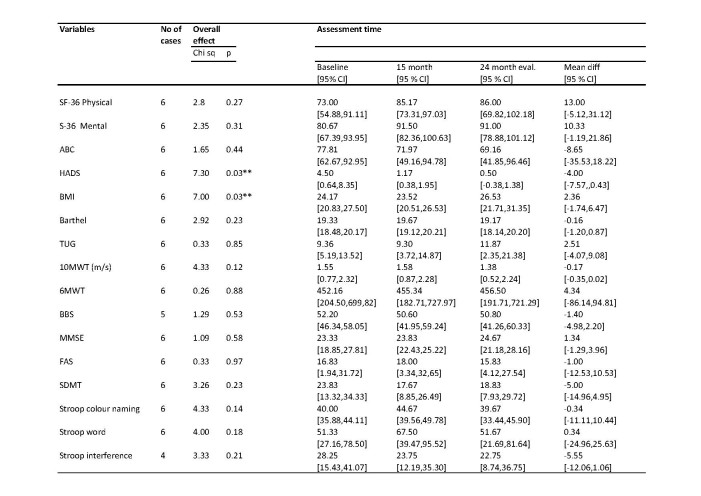
Table 1. Mean change in quality of life, cognitive functions, ADL function, BMI, balance and anxiety and depression from baseline to evaluation at 15 months and 24 months. CI, Confidence Interval; SF,36, Short form ,36; MMSE, Mini Mental State Examination; ABC, Activity Specific Balance Confidence Scale; HADS, Hospital Anxiety and Depression Scale; BMI, Body Mass Index; TUG, timed up and go; 10MWT, 10 meter walk test; 6MWT, 6 minutes’ walk test, BBS, Bergs balance scale; FAS, Verbal Fluency Test; SDMT, Symbol Digit Modalities Test

**Figure:**
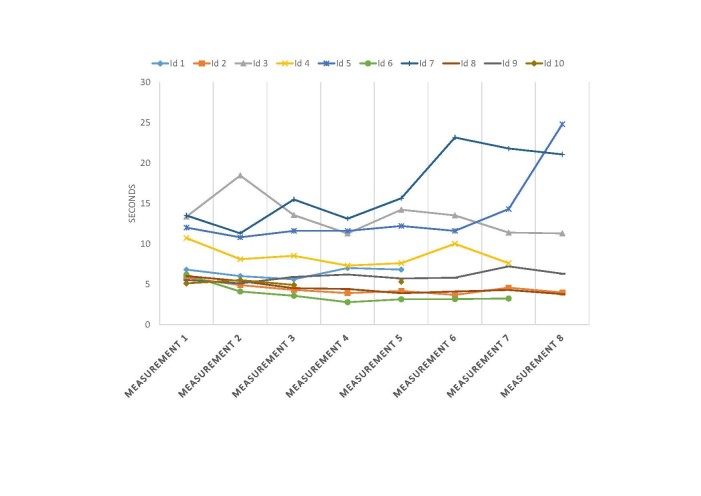
Figure 1. Variation in gait in individual cases measured with “timed up and go test” through eight measurement points (at the time of admission) during two years period.

**Figure:**
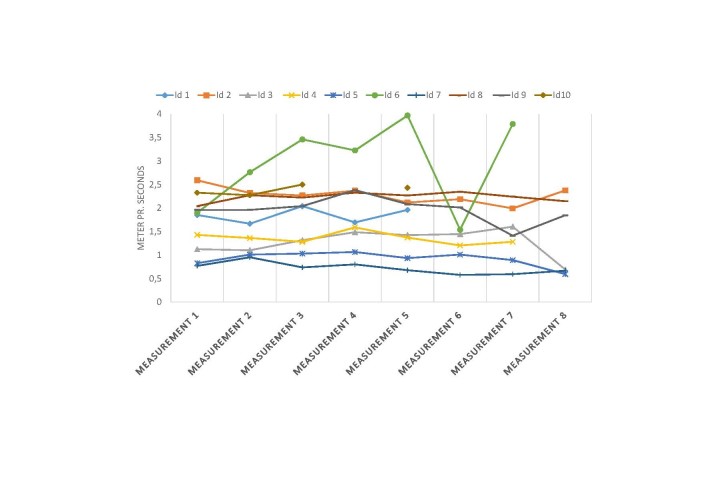
Figure 2. Variation in gait in individual cases measured with “10 meter walk test” through eight measurement points (at the time of admission) during two years period.

**Figure:**
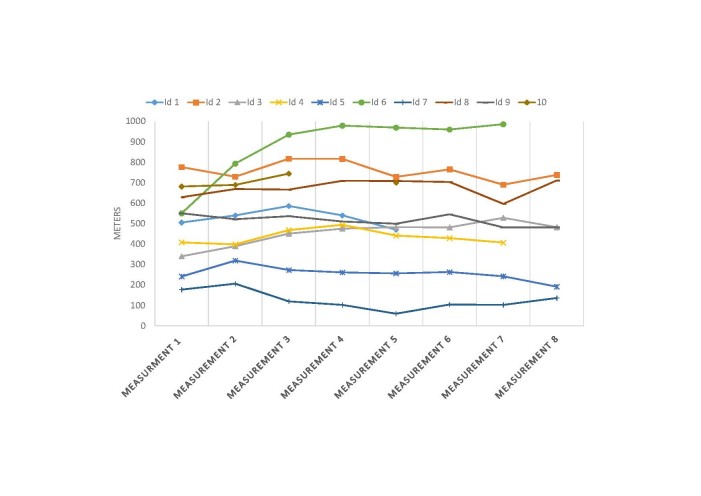
Figure 3. Variation in gait in individual cases measured with “6 minutes' walk test” through eight measurement points (at the time of admission) during two years period.

**Figure:**
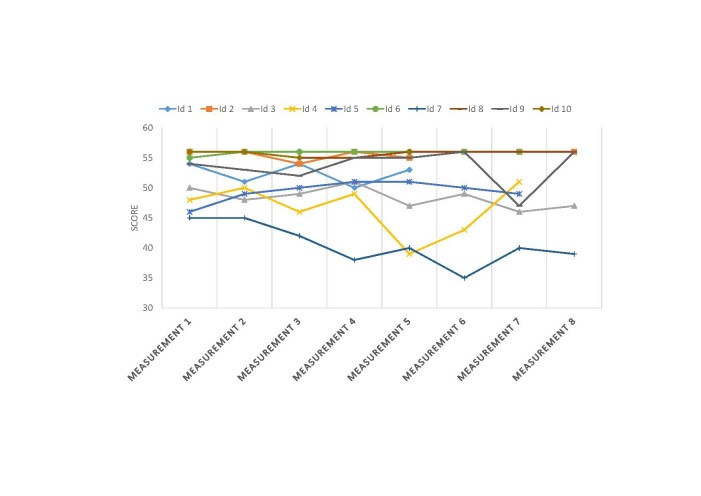
Figure 4. Variation in balance in individual cases measured with “Bergs balance scale” through eight measurement points (at the time of admission) during two years period.

**Figure:**
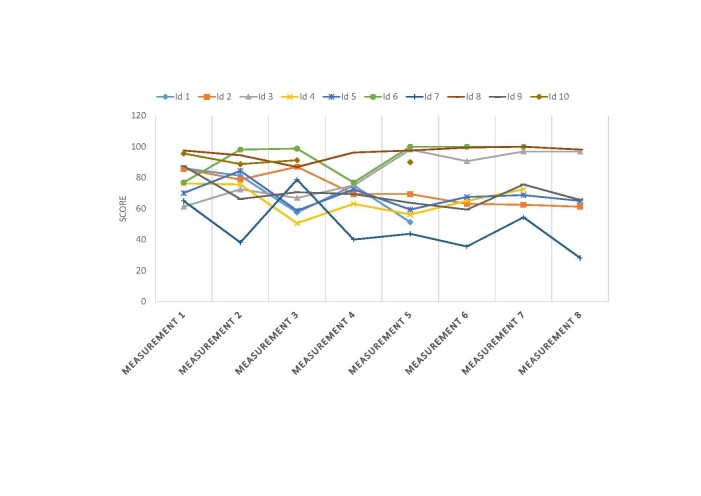
Figure 5. Variation in balance in individual cases measured with “Activities of Balance Confidence scale” through eight measurement points (at the time of admission) during two years period.

**Figure:**
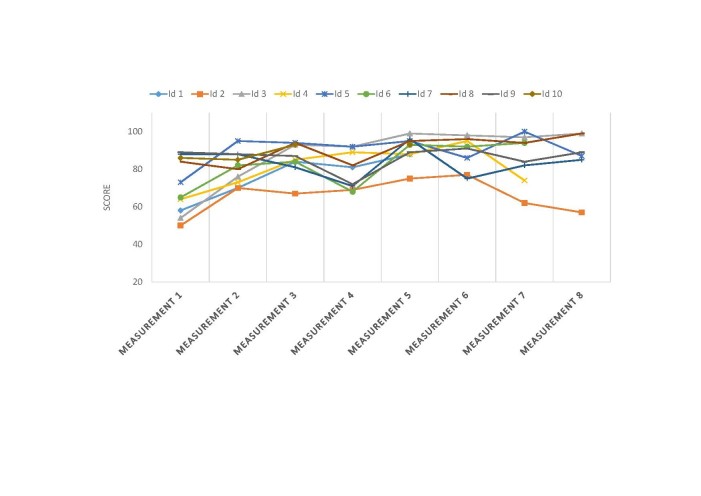
Figure 6. Variation in Physical part of Quality of Life in individual cases measured with “SF-36” through eight measurement points (at the time of admission) during two years period.

**Figure:**
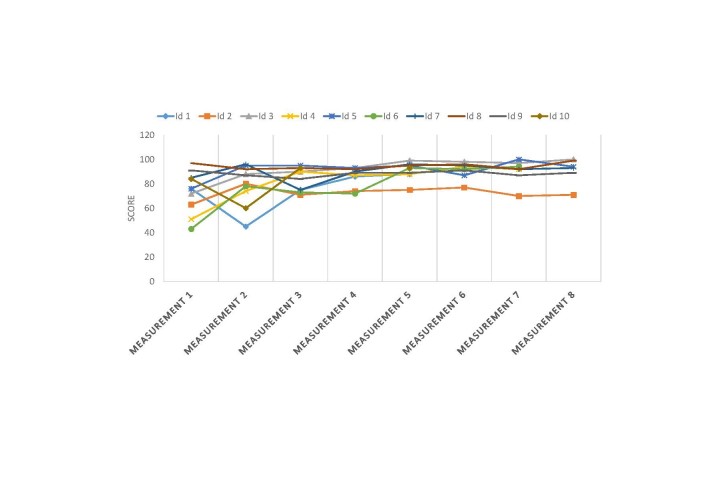
Figure 7. Variation in mental part of Quality of Life in individual cases measured with “SF-36” through eight measurement points (at the time of admission) during two years period.

## Discussion

*Main findings.* The present follow-up study found that six out of ten patients completed the full program. Slight, but non-significant, decline was observed for gait and balance from baseline to the evaluation stay after two years. Non-significant improvements were observed in physical QoL, anxiety and depression, and BMI. ADL-function remained stable with no significant decline. None of the cognitive measures showed a significant decline. An analysis of individual cases revealed that four out of the six participants who completed the program sustained or improved their motor function, while motor function declined in two participants. All the six patients who completed the program reported improved or stable QoL throughout the study period. The participants who were able to complete the two year rehabilitation program showed greater individual variation in function than results at the group level indicated. It seems that individuals with better functional levels at the start, as measured by higher/better scores in gait and balance, are able to sustain achieved results over longer time.

*Participation and drop-out*. Our findings suggest that participation in an intensive rehabilitation program is well tolerated among motivated patients in early- to mid-stages of HD. Six of the 10 patients completed the entire two-year program as planned. We found that dropout rate among participation in an intensive long-term rehabilitation program will increase over time, similarly to what was found in an earlier study by Zinzi et al.^5^ where 29 of 40 (72.5%) patients dropped out. The drop-out rate in our project was lower, four out of 10 (40%), but it must also be noted that the 10 patients who started were recruited from 20 patients who had completed a one-year program, and this group may have had a higher motivation than the other patients. Due to disease-related problems and cognitive impairment, it may be challenging to implement rehabilitation programs for patients with neurodegenerative disorders 21 ^,^ 22. Although it can be challenging to motivate HD patients to participate in programs that extend over a long period of time, the results of our study indicate that it is possible to motivate HD patients in the early- and middle stage of disease to follow an extended structured rehabilitation program.

Since HD patients have complex disabilities, including cognitive challenges, rehabilitation programs require intensive multidisciplinary effort to provide the best possible rehabilitation. Overall, we have positive experiences with the implementation of the rehabilitation program for HD patients^22^. Patients with HD seem to tolerate intensive training/rehabilitation, as long as there is room for individual adjustments. Maintaining the same program structure and familiar surroundings during each admission may be a factor that creates a safe environment. We also think that co-operation with local health care personnel after completion of the program is important for the support of patients in their local municipality. After each stay, a comprehensive medical report was sent to the referring physician and other relevant allied health care personnel, clearly describing the patients’ multidisciplinary needs.

*Methodological considerations*. This study is not a randomized clinical trial, but a descriptive intervention study over a two year period for a small number of participants. Interpretation of our findings should thus be done carefully due to small sample size and lack of a control group. The program included standardized protocols and systematically executed multidisciplinary approaches, which have been carefully planned in terms of use of assessments, measurement points aiming to have the same experienced rater at both baseline and the final evaluation. The assessment for UHDRS motor function and behavior were not conducted at the final evaluation so we have not been able to detect any changes in these scores. Changes in medication were not studied. Whether and how long the observed beneficial effects of intensive rehabilitation can be sustained among HD patients, needs to be assessed with longer follow-up. There is also a need for randomized clinical trials to study the effect of multidisciplinary intensive rehabilitation intervention on progression of HD. Further, it is important to investigate which patients profit most from such intensive rehabilitation.

## Conclusion

Our findings suggest that participation in an intensive rehabilitation program is well tolerated among motivated patients with early to mid-stage HD. The findings should be interpreted with caution due to the small sample size in this study.

## Competing Interests

The authors have declared that no competing interests exist.
